# Potential of *Thuja occidentalis* L. Essential Oil and Water Extracts against Field Crop Pests

**DOI:** 10.3390/molecules29071457

**Published:** 2024-03-24

**Authors:** Janina Gospodarek, Agnieszka Krajewska, Iwona B. Paśmionka, Joanna Bruździńska, Gedyon Tamiru

**Affiliations:** 1 Department of Microbiology and Biomonitoring, University of Agriculture, al. A. Mickiewicza 21, 31-120 Krakow, Poland; iwona.pasmionka@urk.edu.pl (I.B.P.); jbruzdzinska@gmail.com (J.B.); tamiru.menagedyon.sd@student.urk.edu.pl (G.T.); 2 Department of Biotechnology and Food Science, Lodz University of Technology, 90-530 Lodz, Poland; agnieszka.krajewska@p.lodz.pl

**Keywords:** *Sitona* weevil adults, *Aphis fabae* Scop. nymphs and adults, *Leptinotarsa decemlineata* Say adults, ladybug larvae, eastern white-cedar

## Abstract

*Thuja occidentalis* L. essential oil (EOTO) and its compounds, such as terpinyl acetate, bornyl acetate, and β-thujone, are claimed to be highly effective against some storage pests, sanitary insects, or pests of fruit trees, while data about its use in protecting field crops are very scarce. There is also a lack of information in the literature about the insecticidal value of water extracts from *T. occidentalis* (WETOs). Both essential oils (EOs) and water extracts (WEs) from various plants have advantages and disadvantages in terms of their use as insecticides. EOs are generally more effective, but their preparation is more complicated and quite expensive. In turn, WEs are simple to prepare and cheap, but they often have limited effectiveness. Moreover, significant differences in responses exist depending on the species of the donor plant, the method of preparing the extract, its concentration, the species of the pest being controlled, the developmental stage, and even the gender of the pest. The goals of the research were to assess the effect of EOTO and WETOs prepared from dry and fresh matter on the mortality, feeding, and body mass changes of important crop pests, i.e., the black bean aphid, pea leaf weevil, and Colorado potato beetle (CPB), respectively, as well as on the mortality and voracity of non-target organism Asian lady beetle young larvae. EOTO showed significant aphicidal activity with LC_50_ = 0.8267% and 0.2453% after 42 h of the experiment for nymphs and wingless females of black bean aphid, respectively. Adults of CPB were more resistant to EOTO than aphids, with LC_50_ values for females equal to 1.5327% and 1.3113% after 48 h and after 72 h of the experiment. There was no significant effect of EOTO on CPB foraging. Calculated LC_50_ values for pea leaf weevil adults were lower than those for CPB (0.9638% and 0.8573% for males after 12 h and 24 h, respectively). In the case of this pest, a clear reduction in foraging was obtained, with higher concentrations of EOTO resulting in more pronounced reductions in foraging behavior. Concentrations of EOTO above 0.5%, which showed efficacy against the aphid, were lethal to 3-day-old larvae of the Asian lady beetle. WETOs, in turn, showed significant potential in inhibiting adult pea leaf weevil feeding, with very low or no effectiveness in reducing *A. fabae* and CPB, respectively.

## 1. Introduction

Essential oils (EOs) have recently been the subject of much interest among researchers for their potential use as insecticides, among many other applications (veterinary, pharmacology, aromatherapy, production of perfumes) [1,2,3]. Water extracts (WEs), on the contrary, are not as widely researched for use in plant protection. Compared to EOs, they are generally considered less effective [4]. However, their advantages are ease of preparation (no specialized extraction equipment is required), low cost (no need to use much energy for preparation), the possibility of preparing the extract on-site on the farm, and higher safety for beneficial insects than extracts prepared with other solvents or EOs [5]. Many pests quickly become resistant to the active ingredients of synthetic insecticides, and there is a need to constantly search for new active substances [6]. Both EOs and WEs are potential sources of such compounds. In Eos, the presence of some terpenoids and phenylpropanoids is seen as responsible for their repellent and insecticidal activity [7]. The composition of WEs is significantly different than that of EOs [8]. However, some compounds typical of EOs can also occur in WEs [9]. Moreover, plant-based insecticides are a mixture of several compounds, which may slow down the process of building pest resistance. They are also generally safer for the environment, including non-target organisms [10,11,12].

*Thuja occidentalis* L. (Cupressaceae) is a plant native to North America [13], known for its numerous medicinal properties [9,14,15], and used as an ornamental plant in many parts of the world [3,16]. EO extracted from various parts of this plant has been studied for its antimicrobial effects [17,18] as well as its insecticidal effects [19,20,21,22,23,24,25,26]. However, data regarding insecticidal effects mostly refer to storage pests [13,20,21,22,23], sanitary insects [24], or pests of fruit trees [25]. Most of these studies indicate that EO extracted from *T. occidentalis* (EOTO) is highly effective. Much rarer are studies on the effects of EOTO on crop pests. Pavela [26] analyzed the insecticidal properties of 34 EOs from different plants by fumigation against *Spodoptera litorallis* Boisd. larvae and indicated EOTO as highly toxic with LC_50_ ≤ 10 mL/m^3^. Of the three plants in the Cupressaceae family, *Chamaecyparis obtusa* Endl., *Chamaecyparis pisifera* Endl., and *T. occidentalis*, essential oil extracted from *T. occidentalis* showed the strongest insecticidal effect against the green peach aphid, *Myzus persicae* Sulzer [19]. The available literature lacks data on the impact of WETOs (water extracts from *T. occidentalis*) on crop pests.

EOs as complex mixtures revealed neurotoxic activity to pests. It has been proven that the mechanism of repellent or insecticidal action of EOs is related to their interference with the neuromodulator octopamine or with the GABA-gated chloride channel. Octopamine is responsible for many vital functions in insects, such as the development of nervous cells, circadian rhythm, locomotion (“fight or flight”) responses, learning, and memory. Disturbance of this function causes a complete collapse of the pest’s nervous system [7].

The composition of EOTO has been analyzed many times [3,16,27,28]. The main group of constituents identified in EOTO were oxygenated monoterpenes (64.8–87.3%), among which α-thujone was identified in the largest amounts (20.1–61.0%). Other monoterpenoids such as β-thujone (3.6–10.7%), sabinene (3.0–9.3%), and fenchone (4.9–7.7%) were also detected. Some diterpenes were also found in this essential oil, which is a group of terpenoids characteristic of coniferous trees. Byerene was the dominant compound among this group (1.1–12.8%) [16]. The correlation between essential oil composition and plant origin is well known. That is why it should be mentioned that EOTO produced from plant leaves from other areas of the world contained a high amount of myrcene (14.6%), bornyl acetate (9.3%), and terpinyl acetate (8.5%), which occurred in small amounts in oils isolated in plants cultivated in Poland. Compounds of EOTO, such as terpinyl acetate and bornyl acetate, as well as some volatile substances (e.g., β-thujone), are considered responsible for their insecticidal properties [19,29].

The composition of plant extracts is different than that of essential oils and may be solvent-dependent [8]. In the available literature, there is no information about the composition of WETO. WE, from a closely related species of the family Cupressaceae, *Platycladus orientlis* L. (also known as *Thuja orientalis* L.), was richer in polyphenols than the EO of this plant. It also contained significant amounts of flavonoids and tannins. During the extraction of *T. occidentalis* leaves using methanol, components such as alkaloids, glycosides, flavonoids, and tannins are isolated [30]. Tannins are known for their insecticidal effect against many insects, such as *Tribolium confusum* L. [31], *Galleria mellonella* L. [32], and *Amrasca devastans* Distant [33]. These substances were also the main compounds of WEs from *Eucalyptus camaldulensis* Dehnh. and *Salvia officinalis* L. tested against aphids [34]. Polyphenols, in turn, obtained from *Coriandrum sativum* L. and *Anethum graveolens* L., caused high mortality rates in *G. mellonella* larvae [32]. Flavonoids, alkaloids, and glycosides are also mentioned as compounds responsible for the insecticidal activity of plant extracts [35,36,37]. Moreover, some insecticidal active ingredients typical for EOTO could also be extracted with water. Thujone, which is an important compound of EOTO, was extracted from *T. occidentalis* with purified water but in much lower content than it is in EOTO [9]. Thus, we suspect that the content of volatile compounds such as β-thujone, known for its insecticidal properties [29], in WETOs could be low, which may cause its weaker impact on pests than in the case of EOTO.

Black bean aphid (*Aphis fabae* Scop.), pea leaf weevil (*Sitona lineatus* L.), and Colorado potato beetle (CPB) (*Leptinotarsa decemlineata* Say) are among the most dangerous pests of economically important crops such as legumes, potatoes, and beetroot. As a polyphagous species, *A. fabae* can also damage many other plants, both cultivated and wild, always leading to a significant reduction in the quantity and quality of the crop, as well as being responsible for the transmission of plant viral diseases [38]. Plants infested by only 5–10 individuals at an early plant growth stage die 28 days after infestation [39]. *Sitona lineatus,* as an adult form, damages the leaves of Fabaceae plants by eating semicircular areas from the edge of the leaf blade, while its larval form destroys root nodules [40]. The number of pea seeds could be reduced by 18% as a result of the occurrence of only 20 adult pea leaf weevils per m^2^, while 7 larvae per plant resulted in 37% destruction of root nodules [41]. *Leptinotarsa decemlineata*, in turn, leads to the defoliation of potato plants, causing losses estimated to be millions of USD per year. Only in China, potential economic loss as a result of the wider spread of CPB is estimated to be USD 235 million year^−1^ [42], and in Russia, annual loss is estimated as USD 2–2.5 billion [43].

The evaluation of the insecticide’s effectiveness should also include its effects on non-target organisms. The available literature lacks any information on the influence of EOTOs or WETOs on beneficial invertebrates. Some works on the insecticidal use of EOs from various plants that also undertake to evaluate their effects on non-target organisms, such as ladybugs, indicate the possibility of stronger negative effects of EOs on beneficial organisms than on target organisms [5]. The Asian lady beetle (*Harmonia axyridis* Pallas) is a species ranked among the most important natural enemies of the black bean aphid [44].

The research goals were to evaluate the effectiveness of EOTO and WETOs prepared from dry and fresh leaves in limiting black bean aphid nymphs and wingless females, pea leaf weevils, and Colorado potato beetle adults, as well as to check the effect of EOTO on *H. axyridis* young larvae.

We put forward the following hypotheses:

1. The proposed doses of EOTO and WETOs will increase the mortality of the pests mentioned;
2. EOTO and WETOs will reduce the mass of food eaten by CPB and the surface area of leaves eaten by pea leaf weevils;
3. Nymphs and wingless females of *A. fabae,* as well as males and females of CPB and *S. lineatus*, will differ in response to the EOTO and WETOs used;
4. Concentrations effective against aphids will be safe for the non-target organism—*H. axyridis* larvae, i.e., they will not increase the mortality of lady beetles or affect their voracity.

## 2. Results

### 2.1. Black Bean Aphid

Anova revealed a significant (*p* ≤ 0.05) effect of the used concentrations of EOTO primarily on the survival of wingless females of *A. fabae*, while the effect of EO on the survival of nymphs was not significant until 2 days after the use of EO (Tables S1 and S2). Survival of *A. fabae* nymphs decreased significantly after 54 h of the experiment under the influence of the highest concentration of EO tested, i.e., 0.5%, and this effect persisted until 78 h. In contrast, none of the other concentrations significantly affected the survival of nymphs (Table 1). Wingless females showed much higher sensitivity to the EO concentrations used—87% of females died as early as 6 h after using 0.5% EOTO (Table 2), reaching 100% mortality at the 66th hour of the experiment. In comparison, nymphs reached 44% mortality at the same hour of the experiment (66) and at the same concentration (0.5%). A lower concentration of EO—0.2%—also significantly affected wingless females’ survival, causing 27% mortality at 6 h after EO use and nearly 100% mortality at 102 h. The other concentrations, 0.05% and 0.1%, did not significantly affect mortality in this aphid stage. WETOs showed a much weaker effect compared to EOTOs. In the case of nymphs (Table 1 and Table S1), no significant effect was detected. In the case of wingless females, the first effects were achieved only after 54 h—a maximum of 35% mortality under the influence of the extract based on fresh matter in the 20% concentration. After 66 h, mortality in the WETO treatments remained at the level of 37–58% (with 8% mortality in the control), and no significant differences were found between the concentrations of the extracts used, neither depending on whether dry or fresh matter was used. Similar trends persisted until the end of the experiment. The weakest WETO effect (not statistically significant for most of the experiment duration) was demonstrated by WETO prepared from fresh matter at the lowest concentration (10%) (Table 2).

Calculated LC_50_ values of the EOTO for nymphs were significantly higher than those for wingless females (Table 3). For example, at 42 and 90 h after the use of EO, the LC_50_ for nymphs was 0.8267% and 0.3132%, respectively. For wingless females at the same hours of the experiment, the values were 0.2453% and 0.0713%. As time passed after the EOTO application, the LC_50_ values decreased.

### 2.2. Colorado Potato Beetle

**Figure 1:**
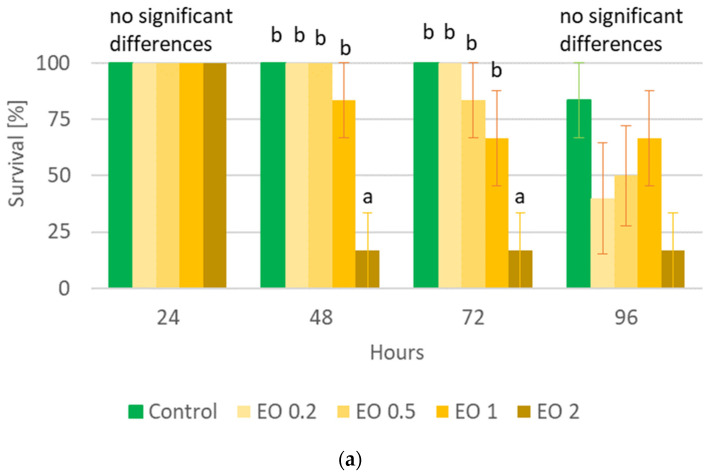

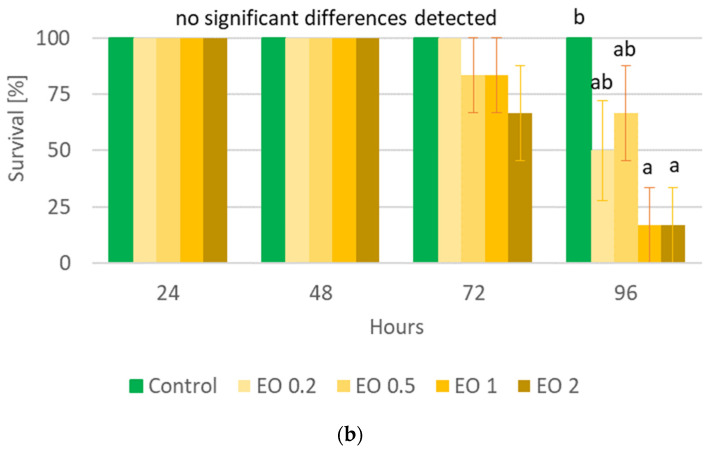
Percentage of live *Leptinotarsa decemlineata* Say females (**a**) and males (**b**) after the application of EOTO. Control—redistilled water; EO 0.2—EOTO in the concentration of 0.2%; EO 0.5—EOTO in the concentration of 0.5%; EO 1—EOTO in the concentration of 1%; EO 2—EOTO in the concentration of 2%. The means for individual dates of observations marked by different letters are statistically different (*p* ≤ 0.05). Where letters are not presented, no significant differences were found.

**Figure 2:**
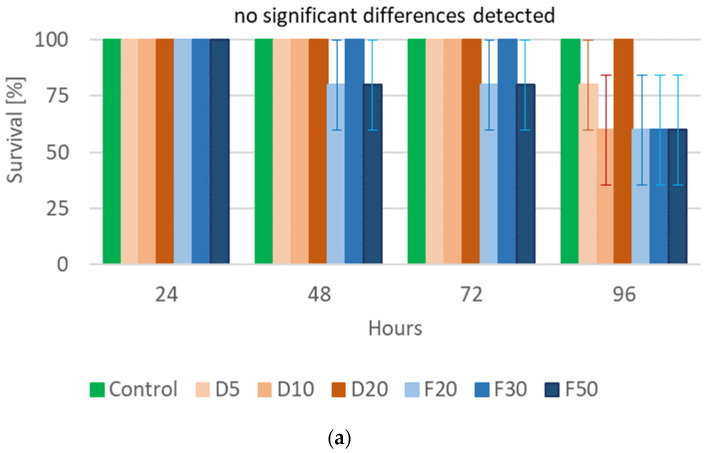

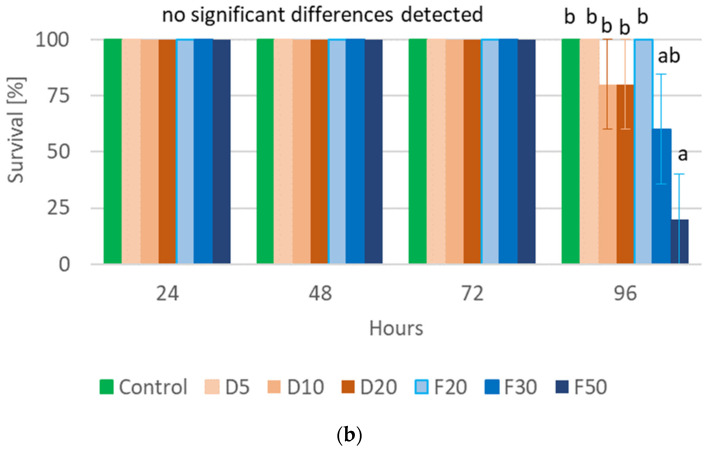
Percentage of live *Leptinotarsa decemlineata* Say females (**a**) and males (**b**) after the application of WETOs. Control—redistilled water; D5—WETO from dry matter in concentration of 5%; D10—WETO from dry matter in concentration of 10%; D20—WETO from dry matter in concentration of 20%; F20—WETO from fresh matter in concentration of 20%; F30—WETO from fresh matter in concentration of 30%; F50—WETO from fresh matter in concentration of 50%. The means for individual dates of observations marked by different letters are statistically different (*p* ≤ 0.05). Where letters are not presented, no significant differences were found.

The performed statistical analysis showed a significant effect of EOTO on the survival of females and males of CPB (Tables S3 and S4), with the effect against males becoming apparent later, i.e., only at the 96th hour of the experiment. Only the highest concentration of EO used—2%—resulted in increased mortality of females (83% of dead insects 48 h after EO use) (Figure 1a). Similar levels of male mortality were obtained using 1% and 2% EO, but only after 96 h of the experiment (Figure 1b). The calculated LC_50_ values for females after 48 h and after 72 h of the experiment were 1.5327% and 1.3113%, respectively (Chi-square value (22 df) = 21.6618 (*p* = 0.4802) and 14.6064 (*p* = 0.8786)). The data obtained regarding the mortality of males at each observation date and females after 96 h of the experiment did not allow for the calculation of reliable values of LC_50_. There was no significant effect of WETOs on female survival (Table S3, Figure 2a). In the case of males, only the extract from fresh matter in the highest concentration (50%) caused a significant increase in mortality after 96 h of the experiment (Table S4, Figure 2b).

Neither EOTO nor WETOs significantly affected the mass of leaves eaten by females of *L. decemlineata* (Table S5, Table 4 and Table 5). In the case of males, there was even an increase in the mass of leaves eaten under the influence of 1% EO after 72 h and 96 h of the experiment (Table 4, Table S6). There was also no significant effect of EOTO and WETOs on the body weight change in CPB adults (Table S7, Table 4 and Table 5).

### 2.3. Pea Leaf Weevil

**Figure 3:**
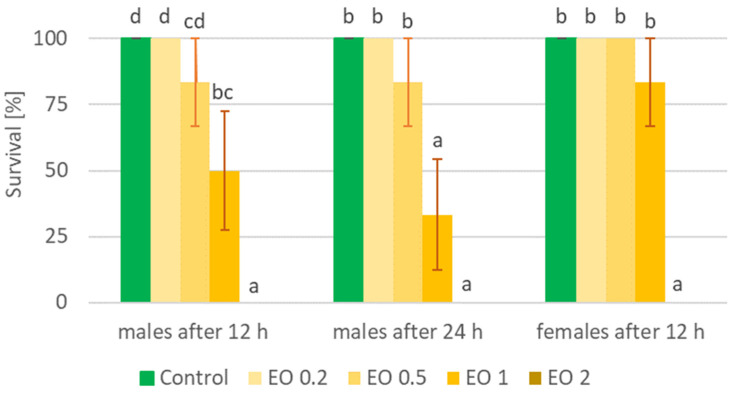
Percentage of live *Sitona lineatus* L. females and males after the application of EOTO. For treatment descriptions, see Table 2. Means ± SE for individual hours (for males and females separately) marked by different letters are statistically different (*p* ≤ 0.05).

The tested EOTO significantly affected the survival of males and females of *S. lineatus* (Figure 3, Tables S8 and S9). A concentration of 2% caused 100% insect mortality in the first hours of the experiment. A concentration of 1% also caused a significant reduction in survival—for males, it was 50% and 66% after 12 and 24 h, respectively, while for females, it was 83% after 12 h. A concentration of 0.5% also caused an increase in mortality in *S. lineatus* males, but the differences with respect to the control group of insects were not statistically proven. The effect of EOTO on the mortality of *S. lineatus* became apparent only at the beginning of the experiment, and then, up to 108 h of observation, there was no reduction in the survival of these insects. In view of the obtained data on beetle mortality, it was not possible to calculate LC_50_ values for *S. lineatus* females. LC_50_ values for males after 12 h and 24 h were 0.9638% and 0.8573%, respectively (Chi-square value (22 df) = 15.4383 (*p* = 0.8428) and 14.2883 (*p* = 0.8909)). In contrast, WETOs did not cause the death of *S. lineatus* beetles.

EOTO significantly affected the surface area eaten by males and females of *S. lineatus* (Table 6, Tables S10 and S11), with the effect being more pronounced for males. Additionally, 1% EO and 0.5% EO caused a significant reduction in the area of damage caused by males compared to the control treatment for virtually the entire duration of the experiment. At the 108th hour of the experiment, the area of leaves treated with 0.5% and 1% EO eaten by males was more than 3-fold and more than 36-fold smaller than that of leaves treated with water, respectively. In contrast, in the case of females, 1% EO significantly reduced the area of the leaves eaten only after 60 h of the experiment. During the last measurement (i.e., after 108 h), there was an approximately 3.5-fold reduction in the area of damages by females in the 1% EO treatment compared to the control. Moreover, 0.5% EO did not significantly reduce the area of damage caused by females.

WETOs caused a significant reduction in the feeding of *S. lineatus* males after 36, 48, and 72 h of the experiment. Later, up to 108 h into the experiment, the differences were not significant (Table 7 and Table S10). WETOs in concentrations of 5% of dry matter and 20% of fresh matter did not show any significant effect. After 72 h, 10% and 20% WETOs from dry matter and 30% and 50% WETOs from fresh matter caused an approximately 4-fold reduction in the surface area eaten by males. The WETO effect on female feeding was statistically significant throughout most of the experiment, with no clear differences depending on the type of extract and its concentration (Table 7 and Table S11). After 108 h of the experiment, WETOs in the lowest concentrations, both from dry (D5) and fresh (F20) matter, did not significantly affect the size of leaf losses, while the remaining WETOs caused a reduction in the eaten area between 2-fold and 6-fold, depending on the treatment.

**Figure 4:**
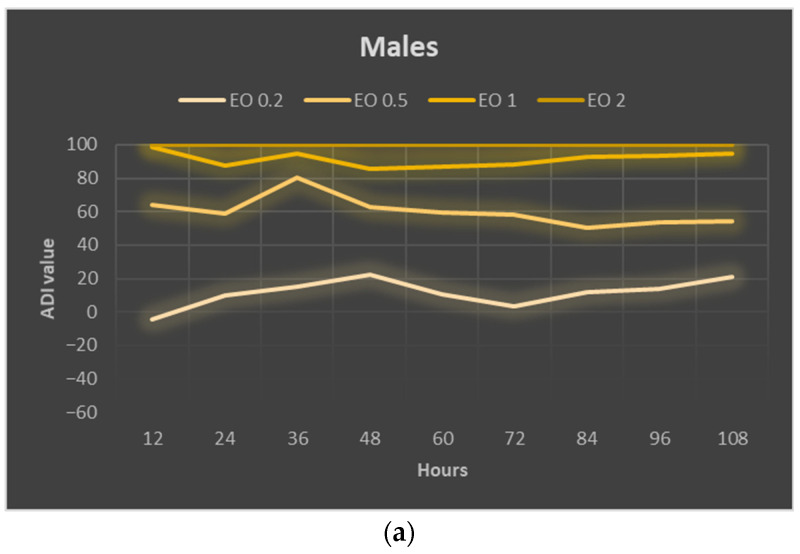

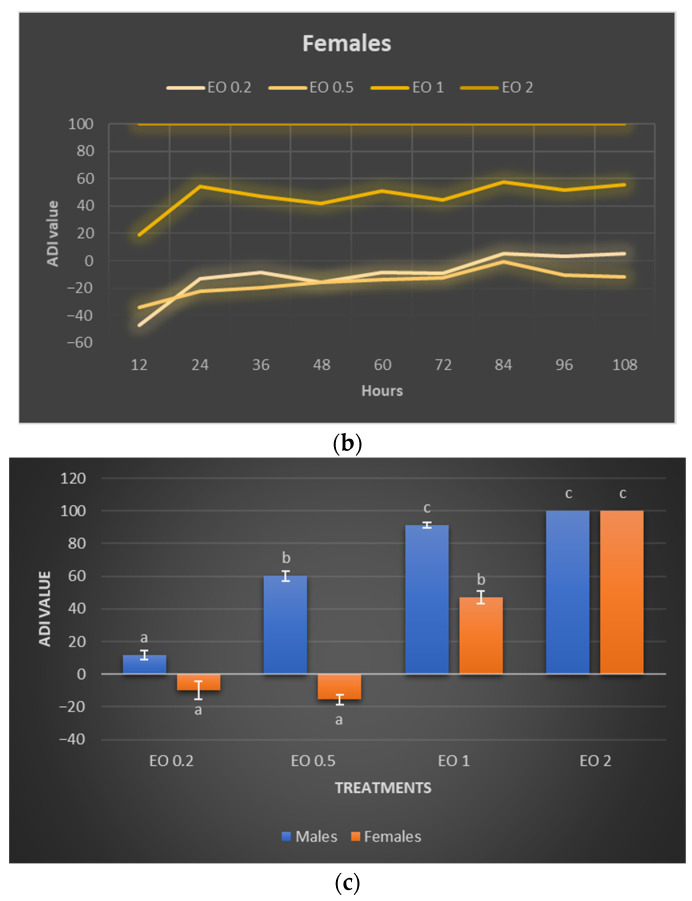
Absolute deterrence index (ADI) of EOTO for males (**a**) and females (**b**) of *Sitona lineatus* L. at specific hours of the experiment and the mean ADI for all dates of observations (**c**). For treatment descriptions, see Table 4. Means ± SE that are marked by different letters in (**c**) (for males and females separately) are statistically different (*p* ≤ 0.05).

The calculated ADI index for males reached positive values in almost every case, indicating the inhibitory effect of the used EOTO concentrations on their foraging (Figure 4a). The values of this index were higher the higher the concentration of EOTO and remained at a similar level in a given treatment throughout the experiment. The deterrent effect of EOTO in the case of females became apparent only at higher concentrations, i.e., 1% and 2%. EOTO concentrations of 0.2% and 0.5% even caused ADI to reach negative values during the initial period of the experiment, indicating an increase in the attractiveness of food treated with these EOTO concentrations. Over time, however, this effect faded. A comparison of the two sexes for 1% EOTO shows significantly higher ADI values for males (between 85 and 98; average 91) than for females (between 18 and 57; average 47) (Figure 4a–c). EOTO, at a concentration of 2%, caused complete inhibition of foraging in both females and males. Analysis of mean ADI values (Figure 4c, Table S12) confirmed the trends described above, i.e., significant differences between concentrations of 0.2%, 0.5%, and 1% for males and no significant differences between treatments where 0.2% and 0.5% EOTO were used for females.

**Figure 5:**
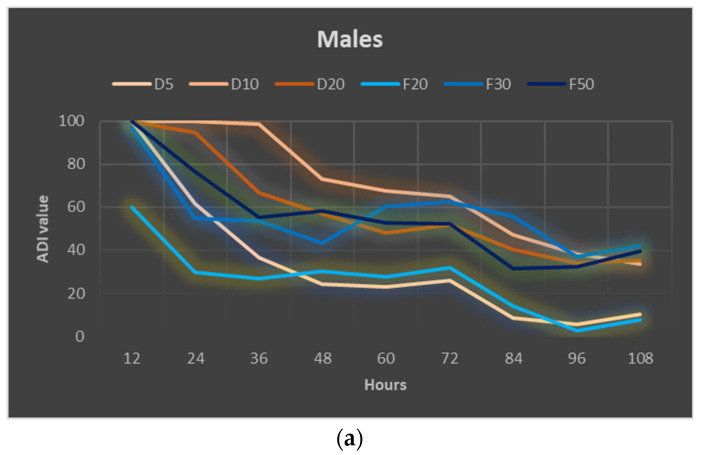

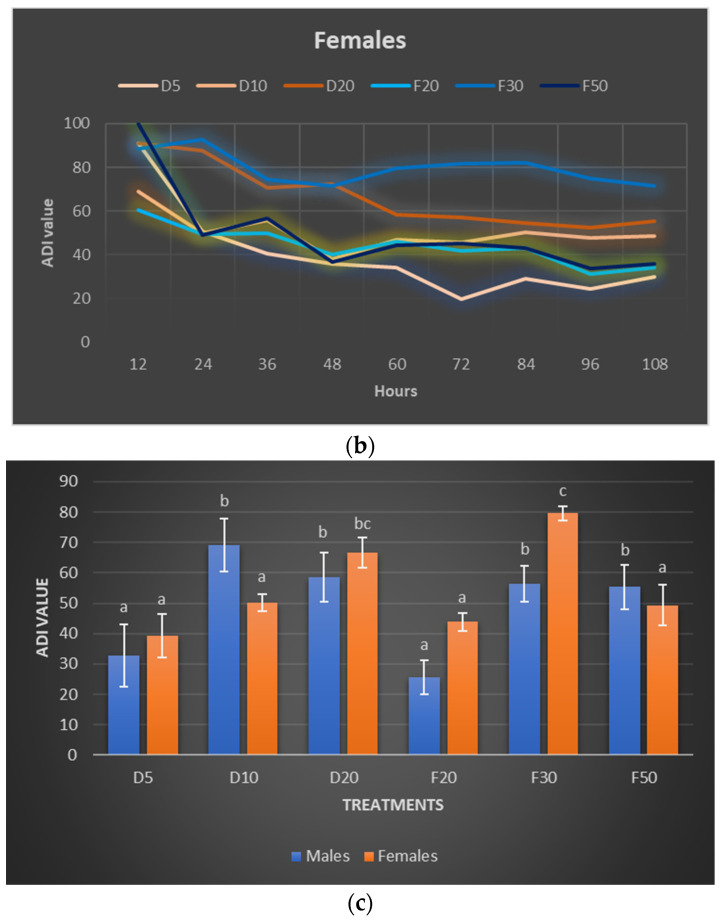
Absolute deterrence index (ADI) of WETOs for males (**a**) and females (**b**) of *Sitona lineatus* L. at specific hours of the experiment and the mean ADI for all dates of observations (**c**). For treatment descriptions, see Table 5. Means ± SE that are marked by different letters in (**c**) (for males and females separately) are statistically different (*p* ≤ 0.05).

The ADI index calculated for WETOs reached positive values for both males and females throughout the whole experiment (Figure 5a,b). In the case of males, the deterrent effect weakened over time, while in the case of females, it generally remained at a similar level throughout the experiment (except for WETO from fresh matter in the highest concentration—50%, which caused complete inhibition of feeding, but only for the first 12 h). A comparison of average values from the entire experiment showed that in the case of WETOs from dry matter, the higher the concentration of the extract, the higher the degree of inhibition of female feeding, while in the case of WETOs from fresh matter, the concentration of 30% was the most effective (Figure 5c, Table S12). In the case of males, no significant differences in the ADI value were observed between the higher doses of WETOs (10% and 20% of dry matter and 30% and 50% of fresh matter).

### 2.4. Asian Lady Beetle

**Figure 6:**
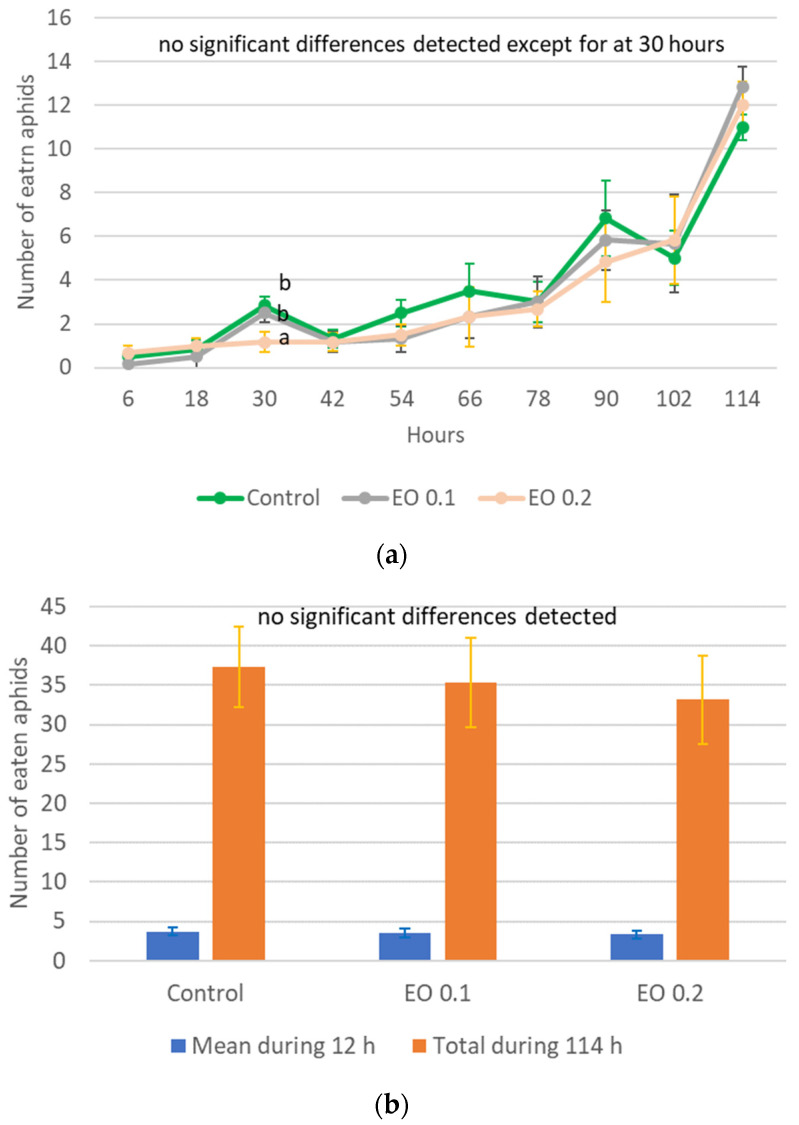
Number of aphids eaten by one 3-day-old larva of *Harmonia axyridis* Pallas in 12 h intervals after the application of EOTO (**a**); mean and total values of the number of eaten aphids (**b**). Control—redistilled water; EO 0.1—EOTO in the concentration of 0.1%; EO 0.2—EOTO in the concentration of 0.2%; Means ± SE for individual dates of observation marked by different letters are statistically different (*p* ≤ 0.05). Where letters are not presented, no significant differences were found.

*Harmonia axyridis* was exposed only to EOTO due to the fact that the afficidal effect of WETOs in the experiment was negligible. The 3-day-old larvae of *H. axyridis* died 100% at concentrations of 0.5% and 1% of EOTO, while concentrations of 0.1% and 0.2% did not cause larval death. For most of the observation period, there was no significant effect of 0.1% and 0.2% EO on the voracity of ladybugs (Figure 6a and Table S13). Only at 30 h of the experiment, 0.2% EO caused a significant reduction in the number of eaten aphids. However, this did not significantly translate into the total number of aphids eaten by individuals during the 114 h experiment or the average value from the 12 h intervals (Figure 6b, Table S13).

### 2.5. Chemical Composition of EOTO

The oil was analyzed using the GC–MS method. EOTO composition analysis revealed over 40 different volatile constituents, which is 99.1% of the total compounds in this oil (Table 8). The main group was oxygenated monoterpenoids (70.7% in total). Other groups, such as monoterpene hydrocarbons (28.0% in total) and diterpenes (0.5% in total), were also detected. No oxygenated sesquiterpenes were identified in the oil. Sesquiterpene hydrocarbons and diterpenes were represented by only two compounds: δ-cadinene (<0.05%) and beyerene (0.5%). The main component of the oil that was present in the largest quantities was α-thujone (38.5%). Among all monoterpenoids, sabinene (12.9%), fenchone (9.3%), β-thujone (4.9%), terpien-4-ol (7.3%), bornyl acetate (4.3%), and α-terpinyl acete (1.0%) were also identified in significant amounts. The EOTO yield was 1.2%.

## 3. Discussion

### 3.1. Chemical Composition of EOTO

Using the GC–MS method, over forty different volatile constituents were detected in *T. occidentalis* EO in this experiment. The main group of volatile compounds was oxygen derivatives of monoterpenes such as α-thujone (38.5%), fenchone (9.3%), terpien-4-ol (7.3%), β-thujone (4.9%), and bornyl acetate (4.3%). Sabinene (12.9%), a monoterpene hydrocarbon, was also identified in significant amounts. The qualitative composition of most constituents complies with the literature data. Some differences were also observed, such as the higher content of sabinene, terpien-4-ol, and bornyl acetate [16,45,46]. Keita et al. [13] identified 22 EO components from *T. occidentalis*, with α-thujone (49.60%), fenchone (14.03%), and β-thujone (9.05%) being dominant, as in the present experiment. Bornyl acetate was identified at 2.48%, while sabinene was identified at 3.95%. In contrast, a study by other authors [19] identified 16 constituents, including bornyl acetate, at a concentration of 9.31%. Ingredients such as bornyl acetate and α-terpinyl acetate, which were identified in EOTO, are assumed to be responsible for the insecticidal effect against *M. persicae* [19]. The fumigation activity of these two components was stronger than that of other monoterpene compounds ((+)-2-carene, (R)-(+)-limonene, 1-decene, and p-cymene). However, EOTO’s contact activity was slightly stronger than that of terpinyl acetate and bornyl acetate alone. EOTO in our experiment contained lower amounts of these two components than reported in [19], but still significant (bornyl acetate—4.3%, α-terpinyl acetate—1.0%). It was proven before that the components of EOs, such as phenylpropanoids (e.g., eugenol, estragole, anethole) and monoterpenoids (e.g., thymol, fenchone), revealed insecticidal and repellent activity [7,47]. From these compounds, fenchone was present in our EOTO in a substantial amount (9.3%). Tested against storage pests *Prostephanus truncatus* (Horn) and *Sitophilus zeamais* Motschulsky fenchone caused 55–80% repellency and prevented *S. zeamais* emergence. However, the crude oil of *Plectranthus glandulosus* Hook., in which fenchone was a major compound, had higher efficiency [47]. Other research also showed a rather weak performance of fenchone against the different stages of *Musca domestica* L. in comparison to another component of *Foeniculum vulgare* L. oil—trans-anethole and oil themselves [48]. According to Wróblewska-Kurdyk et al. [29], volatile compounds such as β-thujone and its derivatives have high repellent activity against *M. persicae,* while α-thujone (the main component of our EOTO) did not affect the settling activity of this aphid. β-thujone was a significant constituent in the analyzed EOTO (4.9%), and we suspect that it could be responsible for the activity of this oil. Moreover, we consider that it may cause essential oils to have higher repellent/insecticidal activity than WETOs. The composition of extracts and macerates is significantly different than that of EOs [9,30]. However, some of the mentioned volatiles can occur in extracts. Because the activity of WETOs was very low, we suspect that the volatile composition of this product is irrelevant. Other compounds of EOTO that showed significant insecticidal activity in experiments conducted by Song et al. [19], such as limonene and *p*-cymene, were also identified in our EOTO, but in small amounts. Summarizing, of the many substances with proven insecticidal properties found in the tested EOTO, the following ones had a significant share: β-thujone, bornyl acetate, and α-terpinyl acetate, and they are probably responsible for the observed insecticidal and deterrent effects. Moreover, EOTO is a mixture of many ingredients that can interact synergistically and enhance the effect of EOTO compared to its single components, as has already been observed in other studies [19,47,48].

The yield of EOTO isolated from fresh leaves of different varieties of *T. occidentalis* collected in Poland fluctuates from 0.1 to 1.5% [16], which is similar to the yield obtained in our experiment (1.2%). Other authors reported an EOTO yield of 0.35% [19].

### 3.2. Black Bean Aphid

From four concentrations of EOTO (0.05%, 0.1%, 0.2%, and 0.5%), which were assessed against nymphs and wingless females of *A. fabae*, concentrations of 0.05% and 0.1% did not show any significant effect on the survival of the black bean aphids. In contrast, a concentration of 0.2% caused a significant increase in mortality, but only in wingless females. Only the concentration of 0.5% caused significant mortality in females of *A. fabae* already after 6 h of the experiment (87%) and moderate mortality in nymphs (36% after 54 h). Hence, the calculated LC_50_ values for wingless females were significantly lower than for nymphs (0.2453% and 0.8267% after 42 h of the experiment for wingless females and nymphs, respectively). In the available literature, there is no information on the use of EOTO against black bean aphids, and data on its use against other aphid species are very scarce. Contact activity (by spraying) of EOTO at concentrations of 5%, 10%, 15%, and 20% with surfactant (8% Tween 20) against peach aphid nymphs was higher than when using individual components of this EO, i.e., terpinyl acetate and bornyl acetate, causing the death of 95% of aphids in 60 min with 10% EO [19]. In contrast, using EOTO as a fumigant, the same authors achieved 100% mortality at a concentration of 5.0 μL/L after 18 h. The concentrations used in the cited experiment with contact action were thus much higher than those used in our experiment.

WETOs were found to be significantly less effective in limiting *A. fabae* than EOTOs. There was no significant effect of any of the WETOs used, regardless of the concentration or the type of material used to prepare the extract, on the nymphs of black bean aphids. The mortality of wingless females increased under the influence of WETOs only after 54 h of the experiment (15–35% mortality in treatments with WETOs). For comparison, after the same time of using EOTO at concentrations of 0.5% and 0.2%, the mortality rate was 94% and 60%, respectively. Comparison of the effectiveness of WETOs with WEs from other plants indicates their rather weak aphicidal properties (25% WE from dry *Thymus algieriensis* Boiss. & Reut. caused 70% mortality of *A. fabae* nymphs after 24 h [4]; *Artemisia dracunculus* L. WEs caused 61–71% and 50–78% mortality of wingless females and nymphs of *A. fabae*, respectively, after 96 h, with only 33% and 26% mortality of these stages in control [49]. However, there are WEs from different plants that did not show any aphicidal properties against *A. fabae* [4].

Comparing the calculated LC_50_ values for the contact activity of EO from different plants against aphids, it seems that EOTO has sufficient efficiency to be indicated as an effective aphicide. In addition, the widespread availability of plant material [9] and the high yield of EO obtained should be emphasized [16]. In comparison, the LC_50_ calculated for nymphs and wingless females of the black bean aphid for peppermint *Mentha piperita* L. EO after 30 h was 0.3400% and 0.3375%, respectively [50]. The same oil used against *Aphis punicae* Passerini showed LC_50_ after 24 h = 2.971 μg/mL [51], while being used as a nanoemulsion against a cotton aphid resulted in LC_50_ of 3879.5 μL of active ingredient/L [52]. The cited values are similar to those obtained in the present experiment. The literature indicates LD_50_ below 1 μL (μg) mL^−1^ as the limit for considering a given EO as an effective insecticide in contact treatment [1], but the type of solvent used and the conditions of the experiment conducted are important. The slightly higher LC_50_ values obtained in the present experiment may be due to the way the target solution was prepared—96% ethanol was used for the initial dilution of the EO, while further dilutions were prepared using redistilled water, which may have somewhat reduced the effectiveness of the EO solutions. Moreover, in the present experiment, only the leaves of the host plant were exposed to the EO solutions, not the aphids themselves, which may also explain the LC_50_ > 0.1% obtained. In most experiments where the contact effect of EOs is studied, they are applied directly to the body of the insect [1].

Noteworthy is the fact that wingless females proved to be a more sensitive stage to EOTO than nymphs. Previous studies on the effects of plant extracts (both EOs and aqueous extracts) on different aphid stages have shown both higher sensitivity of nymphs [53,54], no differences between the sensitivity of adult forms and nymphs [55], and, as in the present experiment, higher sensitivity of adult forms [50,56]. Higher sensitivity of wingless females than nymphs of *A. fabae* was recorded in studies using peppermint EO, but only at the initial time point of the experiment, i.e., between 0 and 30 h (0.5442% and 0.3768% after 6 h for nymphs and wingless females, respectively) [50]. Later—after 54 h—both LC_50_ values were similar (0.2705% and 0.2807% for nymphs and wingless females, respectively) and corresponded to the LC_50_ calculated for wingless females in the present experiment. Differences between developmental forms in sensitivity to toxic substances may be related to the specific physiological and biochemical processes responsible for the inactivation of xenobiotics, which are often different in different developmental forms of insects [57].

During our experiments involving aphids, a decrease in their survival rate in the control treatment could be observed in the later hours of the experiment (especially above 90 h). It should be emphasized that these experiments with aphids were conducted for a relatively long time. Researchers generally conduct this type of experiment for 24 h, rarely longer [19,58]. The extended observation period caused aphids in the control to die as a result of the natural decomposition of the leaf of the host plant on which the aphids were feeding. At this point, a slightly higher (however not statistically significant) survival rate of nymphs was found in some treatments with EOTO (EO 0.05, EO 0.1, EO 0.2) compared to the control (which may suggest the beneficial influence of low doses of EOTO on aphid survival). This effect, however, can be attributed to the antiseptic effect of EOTO [3,15], which could slow down the process of natural destruction of the leaf on which aphids fed, thus extending the possibility of using this leaf as a food source. Similar effects (sometimes even better performance under the influence of low doses of plant extract) were also observed in other experiments [55].

### 3.3. Colorado Potato Beetle

The present study showed that 2% EOTO causes the death of 83% of CPB females 48 h after application. A similar effect in the case of males was noted only after 96 h, with 1% and 2% EO. There is no available information on the effect of EO from *T. occidentalis*, as well as from other plants of the Cupressaceae family, on *L. decemlineata*. Results using other EOs indicate that CPB is significantly resistant to EOs [50,59] and that adult forms of CPB are more resistant than larval forms [60]. *M. piperita* EO caused mortality in L2 CPB larvae with LC_50_ values of 0.3449% and 0.2020% after 2 and 3 days of the experiment, respectively, while for older larvae (L4) the LC_50_ value was 0.7289% after 96 h [50]. In the present research, LC_50_ values for females were 1.5327% and 1.3113% after 48 h and after 72 h of the experiment. EOs with acetone as a solvent extracted from *Origanum vulgare* Mill. and *Satureja hortensis* L. caused only 20% mortality of adult CPB after 24 h at concentrations of 8000 and 7000 ppm, respectively [59]. There was also wide variation in the effectiveness of EO against adult CPB from different plant species, even from the same family. EO extracted from *Mentha longifolia* used against adult CPB showed effectiveness with LC_50_ = 3561 ppm (after 24 h), while EO from *Mentha spicata* was ineffective [59]. For EO from lavender (acetone as solvent), the same authors calculated LC_50_ = 4154 ppm. Another study showed that the LD_50_ of peppermint EO for adult CPB was 38 µg (after 24 h; topical application), while lavender extracts obtained by various methods (including hydrodistillation, as in the present experiment) were completely ineffective [60]. Thus, data on the performance of EOs extracted even from the same plant species can be divergent, depending on the extraction method, the application method, the solvent used, or the origin of the plant material. The rather high LC_50_ values found in the present experiment can be justified by the way the experiment was conducted—the use of ethanol followed by water for dilutions, as well as the method of application—exclusively on the food being eaten.

As in the case of *A. fabae*, WETOs showed weaker activity against CPB compared to EOTO. CPB females did not respond significantly to any of the WETOs used, and in the case of males, only the highest concentration (50%) of WETO from fresh matter resulted in an increase in their mortality, with the effect noted only after 96 h. The literature emphasizes the low insecticidal effectiveness of plant WEs towards CPB, although WEs may inhibit the growth of larval stages, especially younger ones (L2) [49].

The concentrations of EOTO tested and different WETOs used did not significantly affect the weight of food eaten or cause changes in the body mass of beetles during the experiment. Similarly, peppermint EO at a concentration of 1% did not significantly reduce the foraging and body mass gain of 4th instar larvae of CPB, although younger larvae (2nd instar) ate significantly less food and grew less under the influence of peppermint EO at a concentration >0.5% [50]. Kostić et al. [61], evaluating the antifeedant properties of 0.5% EO (96% ethanol as solvent) from sage (*Salvia officinalis* L.), showed weaker efficacy of the tested solution against adult CPB than against larvae. Potato leaves treated with EO from sage were eaten by adults only approximately 20% less than untreated ones, and the deterrent effect was shortlived. After 5 days of the experiment, there were no longer any differences between treated and untreated leaves. In the case of larvae of CPB (from L2 to nymph), on the other hand, damages in treated leaves were 35–60% lesser compared to untreated ones.

### 3.4. Pea Leaf Weevil

It should be noted that 2% EOTO caused 100% mortality in *S. lineatus* adults already during the first few hours of the experiment. On the other hand, 1% EO caused the deaths of 50% of males and 27% of females after 12 h of the experiment. The calculated LC_50_ values were lower than for *L. decmlineata* (0.9638% and 0.8573% for males after 12 h and 24 h, respectively), and unlike CPB, females appeared to be more resistant. Data on the effects of plant extracts on *S. lineatus* have focused on WEs [54,56,62] and their deterrent effects, while the substances tested did not affect *S. lineatus* survival. Similarly, WETOs in the present experiment did not cause an increase in mortality in pea leaf weevils. In the present study, 1% EOTO reduced the amount of food eaten by nearly 14-fold for males and 3-fold for females after 60 h, while 0.5% EOTO at the same time reduced the damaged area by about 4-fold for males but did not significantly affect foraging by females. WETOs, on the contrary, after 60 h caused an average 3-fold reduction in the area eaten by females (the higher the reduction, the higher the concentration of the extract in the case of WEs prepared from dry matter), but did not significantly affect the feeding of males. This suggests a gender-dependent response.

In the case of males, the calculated ADI index for each analyzed concentration of EOTO reached positive values and higher with higher concentrations of EO. In the case of females, 0.2% EOTO and 0.5% EOTO resulted in negative ADI values, with the effect particularly pronounced in the initial period after EO application, indicating a lack of deterrent effect. The calculated ADI at 1% EOTO reached average values of 91 and 47 for males and females, respectively. In turn, the maximum ADI values for WETOs were 69 for males (10% dry matter) and 80 for females (30% fresh matter). These are quite high values. In comparison, the maximum ADI values found with WEs from various plants were 68 and 65 for males and females, respectively, for tarragon (20% fresh matter extract) [49]; for tansy *Tanacetum vulgare* L. it was 53 (5% dry matter) for males and 47 (30% fresh matter) for females [54]; for lemon balm *Melissa officinalis* L. ADI values reached 68 for females and 18 for males (10% dry matter) [56]; for peppermint—70 for females (5% dry matter) and 53 for males (30% fresh matter) [55]; for fennel *Foeniculum vulgare* Mill. seeds—70 for males (5%) and 38 for females (20%) [62].

### 3.5. Asian Lady Beetle

Due to the poor aphicidal properties of WETOs, their impact on H. *axyridis* larvae has not been assessed. EOTO in the 0.5% concentration, which exhibited high effectiveness against the aphid *A. fabae*, proved to be lethal to 3-day-old *H. axyridis* larvae. In contrast, lower concentrations (0.1% and 0.2%) did not cause mortality in ladybug larvae, nor did they significantly affect their voraciousness. As with the previous insect studied, there is no information on the effects of EOTO on ladybugs or other predatory insects. Data on the effects of other EOs on ladybugs are disparate. Some indicate that their toxicity toward ladybugs is similar to that toward their prey—i.e., aphids (EO from peppermint against 2- and 5-day-old larvae of *H. axyridis* [50]; EO from pennyroyal, peppermint, basil, and orange (fruit) against *Adalia bipunctata* L. and *Coccinella septempunctata* L. adults [63]). However, other studies indicate higher toxicity against predatory insects than prey (EO extracted from *Thymus capitatus* (L.) Hoffmanns. & Link towards coccinellid predator *Cryptolaemus montrouzieri* Mulsant adults [5]; botanical pesticide developed to combat locust, which contained caraway, wintergreen, and orange peel oils at low concentrations against adults and larvae of the ladybird *C. montrouzieri* [64]). Conversely, some studies have shown a higher toxicity of EOs to aphids than to predatory insects (EOs from *Schizogyne sericea* (L.f.) DC. and *F. vulgare* towards L3 larvae and adults of *H. axyridis* [10,11]; *Satureja intermedia* EO towards *Coccinella septempunctata* L. adults [65]). The effect should, therefore, be considered species-specific.

Peppermint EO did not significantly affect the voraciousness of 2-, 5-, and 8-day-old *H. axyridis* larvae for most of the time the experiment was conducted [50]. Occasionally, a reduction in the number of aphids treated with 0.5% peppermint EO and eaten by 8-day-old ladybug larvae and those sprayed with 0.1% and 0.2% peppermint EO eaten by 5-day-old larvae could be observed, which is consistent with the results of the present study, where only after 30 h of the experiment was there a significantly lower number of aphids eaten by *H. axyridis* larvae that had previously been treated with 0.2% EOTO.

## 4. Materials and Methods

### 4.1. EOTO and WETO Preparation, Chemical Composition Analysis of EOTO, and Experimental Design

Leaves of *T. occidentalis* were gathered at the end of June 2022 in Krakow, southeastern Poland. After drying the plant material (25 ± 1 °C, 5 days), hydrodistillation using a Clevenger apparatus was performed for 3 h. The obtained EOTO was then analyzed using gas chromatography combined with mass spectrometry (GC-FID-MS). A detailed description of the method and equipment used for the chemical analysis of EO is given in the previous paper [50]. The components of EOTO were identified by comparing their mass spectra and calculated retention indexes with those presented in [50,66] and computer libraries: NIST 2011 and MassFinder. To prepare a basic solution of EOTO (which amounted to 10%), 96% ethanol was used as a solvent [67]. To minimize the negative effect of ethanol on test insects (found in previous research [50,60]), final concentrations of EOTO were obtained by dilution of the basic solution with redistilled water. In addition, prior to the experiments, the same dilutions of ethanol in redistilled water were tested for their effects on the test insects and no negative impact was found. Finally, six concentrations of EOTO were examined (0.05%, 0.1%, 0.2%, 0.5%, 1%, and 2%).

To prepare WETOs from dry matter, leaves of *T. occidentalis* collected in a similar way as in the case of EOTO were dried and then ground. For the preparation of WETOs from fresh matter, leaves were harvested for 1 h before preparing the extract. WETOs were prepared by pouring a specific mass of plant material (dry or fresh) with an appropriate amount of cold redistilled water in different proportions, i.e., 2:100, 5:100, 10:100, and 20:100 for dry matter and 10:100, 20:100, 30:100, and 50:100 for fresh matter (for example, 2 g of dry plant material poured with 100 mL of H_2_O for the treatment D2—conventionally assumed as 2%). The extracts were then kept in the dark for 24 h. After this time, they were filtered using filter paper and used immediately in the experiments.

Two sets of experiments were performed: one with EOTO and one with WETOs. Redistilled water served as a control in both sets. All experiments were performed in the laboratory (24 °C ± 1°, daylight) in six replicates.

### 4.2. Insect Treatment

Tested insects (*A. fabae* nymphs and wingless females, CPB females and males, *S. lineatus* females and males, and *H. axyridis* larvae) originated from cultures carried out in the Department of Microbiology and Biomonitoring, University of Agriculture in Krakow. Identification of the developmental stages of the insects and estimation of their age were carried out by careful tracking of the biology of the insects in the cultures. The source of the EOTO and WETO influence in experiments with pests were leaves treated with appropriate solutions and, in the case of ladybugs, also aphids, each time supplemented as food. This approach was used to bring laboratory conditions closer to situations possible in the field, where the insecticide acts mainly through the gastric and contact routes. Leaves of host plants were immersed in individual solutions of EOTO, in specific WETOs, or in redistilled water (control), and after draining off for 3 min, they were placed in Petri dishes (diameter of 9 cm, with ventilation), lined with moist filter paper. *Aphis fabae*, CPB, and *S. lineatus* were exposed to both EOTO and WETOs, while *H. axyridis* was exposed only to EOTO due to the fact that the afficidal effect of WETOs in the experiment was negligible.

#### 4.2.1. Black Bean Aphid

One leaf of mock-orange (*Philadelphus coronarius* L.), prepared in the way described above, was placed per dish, along with 10 individuals (wingless females or 6-day-old nymphs). Concentrations ranging from 0.05% to 0.5% of EOTO, 2% to 5% of WETOs from dry matter, and 10% to 30% of WETOs from fresh matter were tested against *A. fabae.* The mortality of aphids was measured nine times, first after 6 h and then every 12 h.

#### 4.2.2. Colorado Potato Beetle

One leaf of *Solanum tuberosum* L., Bella rosa cultivar, prepared in the way described above, was placed per dish, along with one adult (female or male). Due to the higher resistance of CPB to EOTO and WETOs noted in preliminary experiments compared to aphids, concentrations of 0.2% to 2% of EOTO, 5% to 20% of WETOs from dry matter, and 20% to 50% of WETOs from fresh matter were tested. Weight of eaten food (calculated as the difference between the initial leaf weight and the leaf weight at a specific time of the experiment) and mortality of CPB four times at 24 h intervals alongside insect weight change (each individual was weighed separately at the beginning and at the end of the experiment) were measured.

#### 4.2.3. Pea Leaf Weevil

One leaf of *Vicia faba* L., Bartek cultivar, prepared in the way described above, was placed per dish, along with one adult (female or male). Concentrations of 0.2% to 2% of EOTO, 5% to 20% of WETOs from dry matter, and 20% to 50% of WETOs from fresh matter were tested. The parameter measured was the area of losses in leaves, which are the results of the feeding of pea leaf weevil beetles. The measurement was performed nine times at 12 h intervals, followed by the calculation of the absolute deterrence index (ADI = [(K − T):(K + T)]·100, where K is the average area of leaf losses in control [mm^2^] and T is the average area of leaf losses in specific treatment [mm^2^]).

#### 4.2.4. Asian Lady Beetle

One leaf of mock-orange, prepared in the same way as for other test insects, was placed per dish, along with one lady beetle 3-day-old larva and 15 black bean aphid nymphs of identical size (7-day-old) serving as food (each time prior to the addition of new aphids at time intervals, they were sprayed with the specific EOTO solution or with redistilled water, so the food was the source of the EOTO effect on ladybug larvae). Concentrations of 0.1% to 1% of EOTO were used. The choice of concentration for this predator was guided by the effect of EOTO on the survival of *A. fabae*. The lowest concentration tested for *A. fabae*—0.05%—was abandoned due to its lack of any effect on aphid mortality. Furthermore, 3-day-old *H. axyridis* larvae were selected for the experiments, as previous studies have shown higher sensitivity to younger life stages [50]. The voracity of lady beetle larvae, according to [49], and their mortality at 12 h intervals were measured.

### 4.3. Statistical Analysis

The data were pre-checked for normality (Shapiro–Wilk test with Lilliefors correction) and equality of variance (Levene’s test). The significance of differences between the mean values was tested by one-way ANOVA (STATISTICA 13.0 software). Then, a post hoc Duncan’s test was used at *p* ≤ 0.05. Given the much higher efficiency of EOTO than WETOs in the case of EOTO, LC_50_ values were calculated using StatsDirect software (Ver.3.2.7), according to Finney [68].

## 5. Conclusions

The tested EO from *T. occidentalis* showed significant aphicidal activity (comparable to many EOs extracted from other plant species) with LC_50_ = 0.8267% and 0.2453% after 42 h of the experiment for nymphs and wingless females of *A. fabae*, respectively. Adults of *L. decemlineata* were more resistant to EOTO than *A. fabae*, with LC_50_ values for females equal to 1.5327% and 1.3113% after 48 h and after 72 h of the experiment. At the same time, there was no significant effect of EOTO on *L. decemlineata* foraging. Calculated LC_50_ values for *S. lineatus* adults were lower than those for *L. decmlineata* (0.9638% and 0.8573% for males after 12 h and 24 h, respectively). In the case of this pest, a clear reduction in foraging was obtained in individuals that survived contact with EOTO, with higher concentrations of EOTO resulting in more pronounced reductions in foraging behavior. The effectiveness of foraging inhibition was quite high compared to other plant extracts (the calculated ADI at 1% EOTO reached average values of 91 and 47 for males and females, respectively). Concentrations of EOTO above 0.5%, which showed efficacy against the aphid *A. fabae*, were lethal to 3-day-old larvae of *H. axyridis*. Lower concentrations of EOTO—0.1% and 0.2%, on the other hand—proved to be completely safe to the predator—100% of individuals survived and did not significantly affect the voraciousness of *H. axyridis* larvae.

WETOs were found to be significantly less effective in limiting *A. fabae* than EOTO, and their aphicidal effect was weak compared to WEs from other plants. Even at high concentrations (50% from fresh matter and 20% from dry matter), WETOs did not affect the feeding of adult CPB or the survival of females of this pest, and the survival of males was reduced only at the highest concentration (50% WETO from fresh matter). There was also no significant effect of WETOs on the survival of *S. lineatus*. In contrast, there was a significant inhibition of feeding of adult pea leaf weevils after WETO application, with ADI index values similar to those obtained with EOTO at a concentration of 1%.

The effects of EOTO and WETOs were also pest stage/gender-dependent. Wingless females of *A. fabae* proved to be more sensitive to EOTO and WETOs than nymphs. Males in CPB seem to be more sensitive to EOTOs and WETOs than females. A different response of males and females of pea leaf weevils to the use of EOTO and WETOs was noticed—females showed higher feeding inhibition under the influence of WETOs, while males responded stronger under the influence of EOTO.

Given the widespread availability of plant material for oil extraction and the high yield of the oil, it is possible to point to the use of EO from *T. occidentalis* as a potentially good way to reduce the aphid *A. fabae* and inhibit the feeding of *S. lineatus*. When used against aphids, it is advisable that the concentration of EOTO not exceed 0.5% due to the safety of Coccinellidae. In doing so, it is necessary to conduct phytotoxicity tests beforehand. WETOs, in turn, can be recommended for inhibiting *S. lineatus* feeding. Despite their lower effectiveness, WETOs also seem to be a promising source of active substances, even if not insecticidal, perhaps with a deterrent effect. It seems reasonable to conduct a phytochemical analysis of WETOs in the future and to compare them with commercial insecticides and extracts using other solvents (ethanol and methanol).

## Figures and Tables

**Table 1:** Percentage of live nymphs of *Aphis fabae* Scop. after the application of EOTO and WETOs. Control—redistilled water; EO 0.05—EOTO in the concentration of 0.05%; EO 0.1—EOTO in the concentration of 0.1%; EO 0.2—EOTO in the concentration of 0.2%; EO 0.5—EOTO in the concentration of 0.5%; D2—WETO from dry matter in concentration of 2%; D5—WETO from dry matter in concentration of 5%; D10—WETO from dry matter in concentration of 10%; F10—WETO from fresh matter in concentration of 10%; F20—WETO from fresh matter in concentration of 20%; F30—WETO from fresh matter in concentration of 30%.

Treatment	Hours								
	6	18	30	42	54	66	78	90	102
	EOTO								
Control	100.00 (±0.00) a*	100.00 (±0.00) a	97.50 (±2.50) a	97.50 (±2.50) a	95.00 (±2.89) b	95.00 (±2.89) b	87.50 (±6.29) b	67.50 (±8.54) ab	40.00 (±17.80) a
EO 0.05	98.00 (±2.00) a	94.00 (±4.00) a	94.00 (±4.00) a	92.00 (±3.74) a	92.00 (±3.74) b	84.36 (±2.32) b	74.55 (±3.90) b	72.55 (±3.71) b	50.55 (±16.90) a
EO 0.1	96.00 (±2.45) a	94.00 (±4.00) a	94.00 (±4.00) a	90.00 (±6.32) a	87.78 (±5.82) b	85.78 (±7.46) b	81.78 (±6.57) b	73.78 (±10.21) b	61.78 (±15.84) a
EO 0.2	96.00 (±4.00) a	96.00 (±4.00) a	96.00 (±4.00) a	96.00 (±4.00) a	92.00 (±4.90) b	88.00 (±5.83) b	70.00 (±8.37) ab	48.00 (±12.41) ab	42.00 (±14.63) a
EO 0.5	92.00 (±5.83) a	92.00 (±5.83) a	84.00 (±6.78) a	74.00 (±10.30) a	64.00 (±13.27) a	56.00 (±12.08) a	50.00 (±11.40) a	38.00 (±9.70) a	32.00 (±9.70) a
	WETOs								
Control	100.00 (±0.00) *a	96.67 (±3.33) a	95.00 (±3.42) a	95.00 (±3.42) a	90.00 (±6.83) a	83.33 (±9.19) a	73.33 (±10.85) a	61.67 (±13.52) a	51.67 (±14.24) a
D2	98.33 (±1.67) a	93.33 (±3.33) a	91.67 (±4.01) a	90.00 (±4.47) a	68.33 (±10.46) a	65.00 (±10.57) a	43.15 (±13.94) a	34.44 (±10.53) a	27.59 (±8.98) a
D5	100.00 (±0.00) a	93.33 (±2.11) a	93.33 (±2.11) a	93.33 (±2.11) a	78.33 (±7.03) a	73.33 (±8.43) a	63.33 (±12.56) a	56.67 (±14.06) a	40.00 (±15.49) a
D10	96.67 (±3.33) a	96.67 (±3.33) a	96.67 (±3.33) a	95.00 (±3.42) a	85.00 (±3.42) a	71.67 (±8.33) a	55.00 (±1 4.75) a	50.00 (±12.91) a	41.67 (±13.76) a
F10	95.00 (±3.42) a	88.33 (±3.07) a	88.33 (±3.07) a	85.00 (±4.28) a	78.33 (±4.77) a	70.00 (±8.94) a	60.00 (±9.66) a	46.67 (±8.82) a	35.00 (±10.57) a
F20	98.33 (±1.67) a	96.67 (±2.11) a	93.33 (±2.11) a	91.67 (±3.07) a	76.67 (±8.03) a	70.00 (±10.65) a	65.00 (±11.76) a	51.67 (±10.14) a	40.00 (±12.65) a
F30	96.82 (±2.02) a	86.97 (±3.22) a	85.45 (±3.98) a	82.12 (±3.77) a	75.76 (±6.16) a	62.58 (±8.31) a	54.55 (±9.47) a	44.70 (±8.65) a	34.85 (±7.29) a

* Means ± SE marked by different letters (a, b, ab…) in columns (for EOTO and WETOs separately) are statistically different (*p* ≤ 0.05).

**Table 2:** Percentage of live wingless females of *Aphis fabae* Scop. after the application of EOTO and WETOs. For treatment descriptions, see Table 1.

Treatment	Hours								
	6	18	30	42	54	66	78	90	102
	EOTO								
Control	100.00 (±0.00) b*	100.00 (±0.00) c	98.33 (±1.67) c	98.33 (±1.67) c	96.67 (±3.33) c	93.33 (±4.22) c	64.26 (±6.35) b	49.26 (±8.81) b	35.93 (±9.56) b
EO 0.05	100.00 (±0.00) b	100.00 (±0.00) c	100.00 (±0.00) c	100.00 (±0.00) c	100.00 (±0.00) c	91.67 (±4.77) c	80.00 (±6.83) b	48.33 (±9.10) b	28.33 (±5.43) b
EO 0.1	98.33 (±1.67) b	98.33 (±1.67) c	95.00 (±2.24) c	95.00 (±2.24) c	90.00 (±2.58) c	83.33 (±5.58) c	75.00 (±6.71) b	53.33 (±8.82) b	35.00 (±5.63) b
EO 0.2	71.67 (±18.33) b	65.00 (±19.10) b	55.00 (±19.62) b	51.67 (±18.33) b	39.81 (±14.71) b	29.81 (±16.19) b	21.30 (±13.94) a	8.70 (±5.60) a	3.52 (±2.23) a
EO 0.5	13.33 (±13.33) a	13.33 (±13.33) a	13.33 (±13.33) a	6.67 (±6.67) a	6.67 (±6.67) a	0.00 (±0.00) a	0.00 (±0.00) a	0.00 (±0.00) a	0.00 (±0.00) a
	WETOs								
Control	100.00 (±0.00) a	100.00 (±0.00) a	100.00 (±0.00) a	98.33 (±1.67) a	98.33 (±1.67) c	93.33 (±2.11) b	68.33 (±4.01) c	43.33 (±3.33) c	16.67 (±3.33) b
D2	96.67 (±2.11) a	96.67 (±2.11) a	93.33 (±2.11) a	91.67 (±3.07) a	80.00 (±2.58) b	53.33 (±10.85) a	23.33 (±11.16) ab	8.33 (±4.77) a	1.67 (±1.67) a
D5	98.33 (±1.67) a	95.00 (±3.42) a	95.00 (±3.42) a	88.33 (±3.07) a	76.67 (±4.22) ab	58.33 (±8.33) a	30.00 (±7.30) ab	13.33 (±5.58) a	8.33 (±4.77) ab
D10	100.00 (±0.00) a	98.33 (±2.67) a	96.67 (±2.11) a	90.00 (±2.58) a	73.33 (±7.15) ab	51.67 (±8.72) a	25.00 (±8.47) ab	13.33 (±4.94) a	1.67 (±1.67) a
F10	96.67 (±2.11) a	95.00 (±2.24) a	91.67 (±1.67) a	83.33 (±4.22) a	78.33 (±4.01) ab	60.00 (±12.65) a	46.67 (±12.29) bc	33.33 (±10.22) bc	13.33 (±6.15) b
F20	98.33 (±1.67) a	96.67 (±2.11) a	91.67 (±4.77) a	90.00 (±4.47) a	65.00 (±6.71) a	41.67 (±8.33) a	16.67 (±6.67) a	3.33 (±2.11) a	1.67 (±1.67) a
F30	100.00 (±0.00) a	98.33 (±1.67) a	95.00 (±3.42) a	93.33 (±3.33) a	85.00 (±6.19) bc	63.33 (±6.67) a	31.67 (±9.46) ab	18.33 (±6.54) ab	3.33 (±3.33) a

* Means ± SE marked by different letters (a, b, c, ab, bc…) in columns (for EOTO and WETOs separately) are statistically different (*p* ≤ 0.05).

**Table 3:** The LC_50_ value of the EOTO was calculated for nymphs and wingless females of *Aphis fabae* Scop. at a specific time after treatment.

Life Stage	Hours	LC_50_ (%)	95% Confidence Limits		Slope *	(X^2^) **
Nymphs			Lower	Upper		
	30	1.0989	0.7083	5.8427	1.4417	26.8668 ^1^
	42	0.8267	0.4358	1.8103	1.7420	35.0288
	54	0.6501	0.4485	1.0293	2.1088	34.4596
	66	0.5883	0.4661	0.8729	1.8802	29.8071 ^1^
	78	0.5053	0.3811	0.8452	1.4368	25.3154 ^1^
	90	0.3132	0.1943	0.4843	1.6663	36.3392
	102	0.1050	0.0094	0.5457	0.4781	73.1526
Wingless females	6	0.3237	0.2204	0.4342	6.1580	137.0640
	18	0.3095	0.2043	0.4229	5.9938	143.6515
	30	0.2849	0.1794	0.3996	5.6576	148.4458
	42	0.2453	0.1614	0.3413	7.4098	123.1570
	54	0.2176	0.1342	0.3121	7.5166	147.9525
	66	0.1625	0.1337	0.1948	11.6526	53.5328
	78	0.1376	0.1079	0.1703	9.8867	51.4601
	90	0.0713	0.0302	0.1030	7.4935	36.4395
	102	0.0097	−0.0797	0.0451	6.7504	19.5659 ^1^

* Slope of the regression line, ** Chi-square value (22 df), *p* < 0.05, except for ^1^, where *p* > 0.05. For other dates (6 h and 18 h), according to the collected data on the mortality of nymphs, LC_50_ values were not possible to calculate.

**Table 4:** Mass of leaves eaten by one alive individual of *Leptinotarsa decemlineata* Say (mg) after the application of EOTO and body weight change (mg) after 96 h of exposure. Control—redistilled water; EO 0.2—EOTO in the concentration of 0.2%; EO 0.5—EOTO in the concentration of 0.5%; EO 1—EOTO in the concentration of 1%; EO 2—EOTO in the concentration of 2%; T_0_—body weight of CPB at the beginning of the experiment.

Treatment	Hours				Body Weight Change Compared to T_0_ (mg)
	24	48	72	96	
	Females				
Control	256.27 (±63.59) a*	305.01 (±60.28) a	572.75 (±59.93) a	596.50 (±73.84) a	−60.50 (±5.75) a
EO 0.2	254.06 (±43.50) a	268.88 (±51.16) a	484.26 (±66.09) a	497.24 (±71.77) a	−50.00 (±13.57) a
EO 0.5	282.18 (±32.67) a	282.18 (±32.67) a	605.85 (±72.69) a	664.86 (±67.10) a	−74.80 (±4.85) a
EO 1	216.57 (±52.85) a	245.68 (±54.03) a	687.20 (±109.95) a	779.90 (±121.06) a	−69.60 (±7.35) a
EO 2	297.56 (±63.17) a	310.93 (±0.00) a	474.01 (±0.00) a	474.01 (±0.00) a	−37.00 (±0.00) a
	Males				
Control	227.17 (±48.93) a	254.12 (±53.95) a	557.95 (±43.98) a	606.25 (±48.29) a	−15.33 (±3.42) a
EO 0.2	204.49 (±32.61) a	204.49 (±32.61) a	491.10 (±72.10) a	505.83 (±72.10) a	−18.00 (±4.07) a
EO 0.5	224.89 (±51.48) a	220.58 (±62.82) a	459.62 (±84.54) a	499.30 (±117.30) a	−5.60 (±3.04) a
EO 1	276.17 (±41.96) a	276.17 (±41.96) a	948.69 (±192.21) b	1020.31 (±166.52) b	−39.00 (±21.18) a
EO 2	223.14 (±27.36) a	238.69 (±24.03) a	514.42 (±58.98) a	555.06 (±86.41) a	−16.17 (±3.94) a

* Means ± SE marked by different letters (a, b) in columns (for females and males separately) are statistically different (*p* ≤ 0.05).

**Table 5:** Mass of leaves eaten by one alive individual of *Leptinotarsa decemlineata* Say (mg) after the application of WETOs and body weight change (mg) after 96 h of exposure. Control—redistilled water; D5—WETO from dry matter in concentration of 5%; D10—WETO from dry matter in concentration of 10%; D20—WETO from dry matter in concentration of 20%; F20—WETO from fresh matter in concentration of 20%; F30—WETO from fresh matter in concentration of 30%; F50—WETO from fresh matter in concentration of 50%. T_0_—body weight of CPB at the beginning of the experiment.

Treatment	Hours				Body Weight Change Compared to T_0_ (mg)
	24	48	72	96	
	Females				
Control	282.79 (±35.04) a*	315.54 (±42.55) a	383.25 (±46.81) a	453.01 (±51.56) a	−56.50 (±7.60) a
D5	199.22 (±65.02) a	241.34 (±56.61) a	281.55 (±43.31) a	367.36 (±35.14) a	−33.33 (±4.67) a
D10	224.47 (±66.57) a	279.83 (±65.54) a	330.86 (±70.06) a	383.67 (±84.24) a	−51.00 (±12.29) a
D20	206.30 (±46.25) a	296.58 (±49.27) a	368.88 (±66.91) a	516.57 (±106.84) a	−39.60 (±16.67) a
F20	389.55 (±85.10) a	407.40 (±91.22) a	565.50 (±98.10) a	603.91 (±126.79) a	−27.33 (±6.39) a
F30	340.83 (±63.96) a	376.81 (±42.49) a	400.99 (±30.37) a	444.08 (±20.63) a	−59.67 (±1.45) a
F50	271.97 (±94.64) a	283.04 (±93.00) a	273.47 (±88.29) a	407.50 (±129.42) a	−29.00 (±6.00) a
	Males				
Control	248.05 (±52.35) a	254.70 (±49.63) a	266.01 (±53.76) a	295.88 (±48.87) a	2.25 (±5.98) a
D5	174.16 (±33.25) a	184.95 (±32.50) a	188.44 (±33.09) a	246.32 (±42.31) a	−2.20 (±3.02) a
D10	179.99 (±40.17) a	194.16 (±32.40) a	213.47 (±34.93) a	231.83 (±38.46) a	−12.33 (±6.57) a
D20	212.78 (±97.23) a	216.68 (±99.82) a	235.76 (±109.32) a	260.09 (±123.44) a	2.50 (±5.56) a
F20	260.82 (±24.00) a	261.52 (±22.30) a	270.52 (±27.24) a	295.15 (±38.13) a	−1.40 (±3.01) a
F30	226.99 (±44.51) a	229.99 (±41.51) a	233.18 (±47.72) a	294.83 (±78.61) a	−1.00 (±2.00) a
F50	238.90 (±50.49) a	239.20 (±52.49) a	262.18 (±40.29) a	317.47 (±38.39) a	2.00 (±0.00) a

* Means ± SE marked by the same letter (a) in columns (for females and males separately) means that no significant differences were found (*p* ≤ 0.05).

**Table 6:** The area of the semicircle-shaped losses in leaves [mm^2^/one individual] caused by *Sitona lineatus* L. males and females after the application of EOTO. For treatment descriptions, see Table 4.

Treatment	Hours								
	12	24	36	48	60	72	84	96	108
	Males								
Control	7.76 (±2.82) b*	17.66 (±3.22) c	43.28 (±3.71) b	61.61 (±6.95) b	66.82 (±7.87) c	74.38 (±6.28) b	121.14 (±16.61) c	140.91 (±20.05) c	173.24 (±31.30) c
EO 0.2	8.44 (±4.40) b	14.39 (±5.29) bc	31.73 (±11.03) b	39.25 (±13.09) ab	54.15 (±15.53) bc	69.84 (±21.39) b	95.19 (±30.47) bc	107.21 (±35.00) bc	112.68 (±33.95) bc
EO 0.5	1.70 (±1.55) ab	4.55 (±2.28) ab	4.79 (±2.19) a	14.13 (±4.33) a	16.96 (±3.35) ab	19.78 (±2.75) a	39.85 (±8.03) ab	42.35 (±7.89) ab	51.77 (±9.20) ab
EO 1	0.07 (±0.07) a	1.18 (±0.99) a	1.20 (±0.90) a	4.71 (±1.57) a	4.71 (±1.57) a	4.71 (±1.57) a	4.71 (±1.57) a	4.71 (±1.57) a	4.71 (±1.57) a
EO 2	0.00 (±0.00) a	-	-	-	-	-	-	-	-
	Females								
Control	12.32 (±4.07) ab	49.37 (±12.74) ab	71.25 (±17.54) ab	95.77 (±24.63) ab	128.89 (±35.37) b	145.58 (±36.67) ab	240.85 (±69.66) a	256.24 (±72.09) a	285.58 (±76.95) b
EO 0.2	34.33 (±9.35) c	64.62 (±12.16) b	84.37 (±14.81) b	131.29 (±24.57) b	153.25 (±25.85) b	175.23 (±30.05) b	217.04 (±36.36) a	240.37 (±44.14) a	258.66 (±43.84) b
EO 0.5	24.91 (±5.72) bc	78.00 (±13.67) b	105.42 (±16.70) b	131.58 (±21.27) b	170.11 (±31.14) b	185.76 (±29.89) b	243.83 (±45.49) a	314.28 (±52.46) a	361.30 (±67.13) b
EO 1	8.44 (±3.80) a	14.52 (±5.46) a	25.73 (±11.55) a	39.33 (±16.70) a	41.74 (±14.51) a	55.86 (±20.31) a	65.27 (±20.42) a	82.18 (±26.79) a	81.25 (±21.18) a
EO 2	0.00 (±0.00) a	-	-	-	-	-	-	-	-

* Means ± SE marked by different letters (a, b, c, ab, bc…) in columns (for males and females separately) are statistically different (*p* ≤ 0.05).

**Table 7:** The area of the semicircle-shaped losses in leaves [mm^2^/one individual] caused by *Sitona lineatus* L. males and females after the application of WETOs. For treatment descriptions, see Table 5.

Treatment	Hours								
	12	24	36	48	60	72	84	96	108
	Males								
Control	5.02 (±3.08) a*	29.52 (±18.05) a	62.14 (±17.54) c	73.51 (±15.76) b	119.25 (±42.78) a	145.92 (±40.32) b	160.04 (±40.76) a	168.20 (±37.98) a	210.85 (±34.21) a
D5	0.00 (±0.00) a	6.99 (±4.31) a	28.96 (±12.83) abc	44.90 (±13.21) ab	75.01 (±14.85) a	85.66 (±15.18) ab	134.60 (±25.23) a	151.24 (±30.36) a	171.59 (±30.97) a
D10	0.00 (±0.00) a	0.00 (±0.00) a	0.39 (±0.39) a	11.48 (±5.86) a	23.16 (±7.65) a	31.01 (±5.89) a	57.65 (±10.74) a	74.93 (±16.15) a	104.77 (±27.75) a
D20	0.00 (±0.00) a	0.79 (±0.61) a	12.40 (±5.17) ab	20.09 (±6.33) a	42.05 (±6.88) a	46.46 (±4.98) a	67.78 (±8.79) a	82.53 (±9.48) a	100.40 (±16.19) a
F20	1.26 (±1.26) a	16.01 (±13.31) a	36.02 (±17.58) bc	39.39 (±19.17) ab	67.44 (±35.36) a	75.61 (±40.61) ab	121.09 (±57.43) a	159.63 (±74.91) a	180.94 (±86.94) a
F30	0.08 (±0.08) a	8.56 (±3.45) a	18.76 (±4.88) ab	29.04 (±5.87) a	29.27 (±7.79) a	33.51 (±7.99) a	45.42 (±14.57) a	77.68 (±13.13) a	85.53 (±12.33) a
F50	0.00 (±0.00) a	3.93 (±2.46) a	17.90 (±7.97) ab	19.47 (±7.95) a	36.72 (±17.40) a	45.50 (±19.67) a	83.15 (±30.12) a	86.28 (±32.61) a	91.30 (±30.06) a
	Females								
Control	8.48 (±5.65) a	53.99 (±15.79) b	103.45 (±33.90) a	137.75 (±30.51) b	225.02 (±51.31) b	253.28 (±62.06) b	332.99 (±84.62) b	358.76 (±93.85) a	434.05 (±105.38) b
D5	0.39 (±0.39) a	17.66 (±11.02) a	43.94 (±25.98) a	65.52 (±35.80) ab	111.05 (±48.83) a	169.93 (±68.82) ab	184.01 (±68.28) ab	219.31 (±82.50) a	234.61 (±80.97) ab
D10	1.57 (±1.57) a	18.05 (±6.36) a	29.53 (±7.57) a	61.99 (±21.29) ab	81.56 (±32.63) a	95.29 (±28.45) a	110.64 (±38.24) a	127.51 (±40.48) a	150.61 (±57.65) a
D20	0.39 (±0.39) a	3.53 (±3.03) a	17.66 (±9.45) a	21.98 (±9.44) a	59.65 (±8.37) a	69.07 (±13.23) a	98.08 (±15.64) a	111.80 (±13.22) a	124.73 (±14.57) a
F20	2.09 (±2.09) a	18.32 (±15.28) a	34.54 (±16.32) a	59.12 (±12.69) ab	83.73 (±26.06) a	104.66 (±29.04) a	132.88 (±42.80) a	188.33 (±72.24) a	213.45 (±83.57) ab
F30	0.52 (±0.52) a	2.09 (±1.38) a	15.18 (±6.04) a	23.03 (±8.62) a	25.64 (±10.43) a	25.64 (±10.43) a	32.96 (±14.50) a	51.28 (±23.71) a	72.73 (±35.91) a
F50	0.00 (±0.00) a	18.52 (±7.99) a	28.64 (±8.97) a	64.03 (±19.79) ab	86.76 (±26.10) a	96.17 (±26.60) a	132.56 (±37.20) a	177.80 (±49.76) a	204.51 (±61.21) a

* Means ± SE marked by different letters (a, b, c, ab, bc…) in columns (for males and females separately) are statistically different (*p* ≤ 0.05).

**Table 8:** The chemical composition of EOTO.

No.	Compound	RI_lit_^1^	RI_lab_	EOTO [%]	Lit [%]
1	Methylethyl butyrate		833	0.2	
2	α-Tchujene	926	920	1.0	0–0.7
3	α-Pinene	934	927	2.3	0.2–5.0
4	α-Fenchene	948	938	0.7	0–2.0
5	Camphene	950	939	0.8	0.1–3.0
6	Sabinene	970	964	12.9	1.4–12.0
7	β-Pinene	974	967	0.1	0–1.1
8	Myrcene	983	979	2.3	t–4.1
9	α-Phellenadrene	998	993	0.1	0–0.1
10	Car-3-ene	1006	1001	0.3	0–1.0
11	α-Terpinene	1013	1006	1.7	t–1.8
12	*p*-Cymene	1016	1008	0.6	0.3–2.4
13	β-Phellandrene	1025	1008	0.3	0–1.7
14	Limonene	1025	1018	1.3	0.3–3.2
15	γ-Terpinene	1051	1048	2.7	0.2–2.3
16	*trans*-Sabinene hydrate	1053	1053	0.1	0–1.1
17	Fenchone	1069	1068	9.3	0.4–15.0
18	*p*-Cymenene	1075	1068	t	
19	Terpinolene	1081	1078	0.7	0.2–0.3
20	α-Thujone	1089	1090	38.5	2.8–69.8
21	β-Thujone	1103	1098	4.9	3.1–10.7
22	*cis*-*p*-Menth-2-en-1-ol	1108	1107	0.5	0–0.6
23	Camphor	1123	1119	0.1	0–3.3
24	Neoisothujol	1132	1123	0.3	0.2
25	Sabina ketone	1132	1126	0.1	
26	Camphene hydrate	1143	1131	0.2	0.2
27	Neothujol	1136	1134	0.1	
28	Borneol	1150	1149	0.3	0–0.7
29	Terpinen-4-ol	1164	1163	7.3	1.2–3.3
30	α-Terpineol	1176	1172	0.4	0.1–0.9
31	Estragol	1175	1176	0.1	
32	*cis*-Piperitol	1181	1180	0.1	
33	*trans*-Piperitol	1193	1190	0.1	
34	Fenchyl acetate	1205	1207	2.7	0–1.1
35	Piperitone	1226	1226	t	
36	Linalyl acetate	1239	1240	t	0–1.2
37	Neoisothujan-3-ol acetate		1256	0.1	
38	Bornyl acetate	1270	1270	4.3	0.1–3.9
39	*trans*-Sabinyl acetate	1278	1272	0.1	0–16.6
40	Terpinen-4-ol acetate	1289	1283	0.1	
41	α-Terpinyl acetate	1335	1332	1.0	0–1.8
42	δ-Cadinene	1520	1515	t	0–1.3
43	Beyerene		1936	0.5	0–13.2
	Total identified			99.1	

^1^ RI_lit_—literature retention index; RI_lab_—experimental retention index; EOTO—*Thuja occidentalis* essential oil; Lit—literature data [13,33,34]; t—trace amounts t < 0.05.

## Data Availability

Data are contained within the article and Supplementary Materials.

## Supplementary Materials

The following supporting information can be downloaded at: https://www.mdpi.com/article/10.3390/molecules29071457/s1, Table S1: Statistical analysis on the survival of *Aphis fabae* Scop. nymphs under EOTO and WETOs; Table S2: Statistical analysis on the survival of wingless females of *Aphis fabae* Scop. under EOTO and WETOs; Table S3: Statistical analysis on survival of females of *Leptinotarsa decemlineata* Say under EOTO and WETOs; Table S4: Statistical analysis on survival of males of *Leptinotarsa decemlineata* Say under EOTO and WETOs; Table S5: Statistical analysis on the mass of leaves eaten by one female of *Leptinotarsa decemlineata* Say [g] under EOTO and WETOs; Table S6: Statistical analysis on the mass of leaves eaten by one male of *Leptinotarsa decemlineata* Say [g] under EOTO and WETOs; Table S7: Statistical analysis of body weight change in females and males of *Leptinotarsa decemlineata* Say [g] under EOTO and WETOs; Table S8: Statistical analysis on survival of females of *Sitona lineatus* L. under EOTO; Table S9: Statistical analysis on survival of males of *Sitona lineatus* L. under EOTO; Table S10: Statistical analysis on the surface area of places eaten in leaves by one male of *Sitona lineatus* L. under EOTO and WETOs; Table S11: Statistical analysis on the surface area of places eaten in leaves by one female of *Sitona lineatus* L. under EOTO and WETOs; Table S12: Statistical analysis on the absolute deterrence index for males and females of *Sitona lineatus* L. (mean for all dates of observations) under EOTO and WETOs; Table S13: Statistical analysis on the number of aphids eaten by one 3-day-old larva of *Harmonia axyridis* Pallas under EOTO.
