# Supplementary material for: Potential of Thuja occidentalis L. Essential Oil and Water Extracts against Field Crop Pests
Source: Molecules. 2024 Mar 24;29(7):1457. doi: 10.3390/molecules29071457 (PMC11013141; doi:10.3390/molecules29071457)
Supplement: Supplementary file 1 [file molecules-29-01457-s001.zip › molecules-2892660-supplementary.pdf]

Table S1. Statistical analysis on the survival of *Aphis fabae* Scop. nymphs under EOTO and WETOs.

Hours	Sum of Squares	df	Mean Square	F	p
		EOTO			
6h	162.50	4	40.63	0.643	0.638295
18h	160.00	4	40.00	0.463	0.761716
30h	540.83	4	135.21	1.314	0.300517
42h	1700.83	4	425.21	2.247	0.102172
54h	3118.71	4	779.68	2.930	0.048181
66h	4322.83	4	1080.71	4.173	0.013628
78h	3910.88	4	977.72	3.292	0.032860
90h	5098.15	4	1274.54	2.955	0.046899
102h	2545.61	4	636.40	0.584	0.677806
		WETOs			
6h	121.76	6	20.29	0.731	0.627985
18h	591.38	6	98.56	1.853	0.117141
30h	544.35	6	90.73	1.449	0.224230
42h	901.93	6	150.32	1.954	0.099287
54h	1725.46	6	287.58	0.980	0.453539
66h	1600.79	6	266.80	0.520	0.789441
78h	3288.49	6	548.08	0.633	0.702634
90h	2772.19	6	462.03	0.590	0.735892
102h	1993.27	6	332.21	0.373	0.890834

Table S2. Statistical analysis on the survival of wingless females of *Aphis fabae* Scop. under EOTO and WETOs.
.

Hours	Sum of Squares	df	Mean Square	F	p
		EOTO			
6h	33566.67	4	8391.67	13.535	0.000005
18h	34180.00	4	8545.00	13.052	0.000007
30h	34420.00	4	8605.00	12.568	0.000010
42h	40046.67	4	10011.67	21.484	0.000000
54h	41259.42	4	10314.86	30.858	0.000000
66h	43012.76	4	10753.19	26.835	0.000000
78h	30206.34	4	7551.58	19.286	0.000000
90h	15518.77	4	3879.69	11.999	0.000014
102h	7309.05	4	1827.26	9.668	0.000072
		WETOs			
6h	80.95	6	13.49	1.090	0.387680
18h	123.81	6	20.63	0.774	0.595808
30h	314.29	6	52.38	1.058	0.406083
42h	761.90	6	126.98	1.918	0.105233
54h	3857.14	6	642.86	4.219	0.002695
66h	9447.62	6	1574.60	3.391	0.009635
78h	11123.81	6	1853.97	3.929	0.004182
90h	7328.57	6	1221.43	5.910	0.000244
102h	1400.00	6	233.33	2.988	0.018339

Table S3. Statistical analysis on survival of females of *Leptinotarsa decemlineata* Say under EOTO and WETOs.

Hours	Sum of Squares	df	Mean Square	F	p
		EOTO			
48h	30919.54	4	7729.89	11.131	0.000030
72h	27931.03	4	6982.76	5.586	0.002524
96h	15413.79	4	3853.45	1.623	0.201092
		WETOs			

48h	2857.14	6	476.19	0.833	0.554465
72h	2857.14	6	476.19	0.833	0.554465
96h	10857.14	6	1809.52	0.905	0.505569

Table S4. Statistical analysis on survival of males of *Leptinotarsa decemlineata* Say under EOTO and WETOs.

Hours	Sum of Squares	df	Mean Square	F	p
		EOTO			
72h	6666.67	4	1666.67	1.190	0.339322
96h	30000.00	4	7500.00	4.167	0.010117
		WETOs			
96h	25714.29	6	4285.71	3.333	0.013239

Table S5. Statistical analysis on the mass of leaves eaten by one female of *Leptinotarsa decemlineata* Say [g] under EOTO and WETOs.

Hours	Sum of Squares	df	Mean Square	F	p
		EOTO			
24h	0.02297	4	0.00574	0.347	0.843482
48h	0.01120	4	0.00280	0.200	0.934988
72h	0.11812	4	0.02953	0.925	0.472290
96h	0.23631	4	0.05908	1.588	0.225640
		WETOs			
24h	0.15263	6	0.02544	1.102	0.38583
48h	0.10190	6	0.01698	0.779	0.59317
72h	0.24259	6	0.04043	2.117	0.08551
96h	0.18048	6	0.03008	0.920	0.49694

Table S6. Statistical analysis on the mass of leaves eaten by one male of *Leptinotarsa decemlineata* Say [g] under EOTO and WETOs.

Hours	Sum of Squares	df	Mean Square	F	p
		EOTO			
24h	0.01714	4	0.00428	0.414	0.796584
48h	0.01850	4	0.00462	0.414	0.797031
72h	0.69104	4	0.17276	4.202	0.011206
96h	0.80384	4	0.20096	4.453	0.009207
		WETOs			
24h	0.03307	6	0.00551	0.385	0.882094
48h	0.02493	6	0.00416	0.296	0.933579
72h	0.02755	6	0.00459	0.295	0.934206
96h	0.02954	6	0.00492	0.230	0.963432

Table S7. Statistical analysis on body weight change of females and males of *Leptinotarsa decemlineata* Say [g] under EOTO and WETOs.

	Sum of Squares	df	Mean Square	F	p
		EOTO			
females	0.00246	4	0.00062	1.682	0.20042
males	0.00259	4	0.00065	2.074	0.11881
		WETOs			
females	0.00341	6	0.00057	1.246	0.332143
males	0.00049	6	0.00008	1.012	0.448394

Table S8. Statistical analysis on survival of females of *Sitona lineatus* L. under EOTO.

Hours	Sum of Squares	df	Mean Square	F	p
12h	45333.33	4	11333.33	34.000	0.000000

Table S9. Statistical analysis on survival of males of *Sitona lineatus* L. under EOTO.

Hours	Sum of Squares	df	Mean Square	F	p
12h	43333.33	4	10833.33	11.607	0.000018
24h	48000.00	4	12000.00	13.846	0.000004

Table S10. Statistical analysis on the surface area of places eaten in leaves by one male of *Sitona lineatus* L. under EOTO and WETOs.

Hours	Sum of Squares	df	Mean Square	F	p
		EOTO			
12h	418.85	4	104.71	2.935	0.040593
24h	832.18	3	277.39	3.519	0.039422
36h	5548.77	3	1849.59	6.672	0.004428
48h	8346.91	3	2782.30	5.990	0.006822
60h	10474.14	3	3491.38	5.618	0.008722
72h	14565.80	3	4855.27	4.836	0.015041
84h	30928.00	3	10309.33	4.133	0.025383
96h	43226.23	3	14408.74	4.318	0.022049
108h	62569.44	3	20856.48	4.764	0.015842
		WETOs			
12h	104.39	6	17.40	2.126	0.083044
24h	3146.53	6	524.42	1.312	0.285802
36h	11213.24	6	1868.87	2.817	0.029241
48h	12703.78	6	2117.30	3.013	0.021888
60h	33139.09	6	5523.18	1.990	0.102282
72h	48459.41	6	8076.57	2.725	0.033539
84h	54564.52	6	9094.09	1.789	0.139109
96h	51973.54	6	8662.26	1.199	0.337864
108h	74795.66	6	12465.94	1.421	0.244602

Table S11. Statistical analysis on the surface area of places eaten in leaves by one female of *Sitona lineatus* L. under EOTO and WETOs.

Hours	Sum of Squares	df	Mean Square	F	p
		EOTO			
12h	4451.48	4	1112.87	6.135	0.001393
24h	12040.56	3	4013.52	4.916	0.010775
36h	18261.52	3	6087.17	4.308	0.017717
48h	30267.29	3	10089.10	3.456	0.037066
60h	49713.71	3	16571.24	3.674	0.031747
72h	53376.68	3	17792.23	3.424	0.039513
84h	111606.35	3	37202.12	2.850	0.066391
96h	149103.80	3	49701.27	3.153	0.050323
108h	212346.02	3	70782.01	3.810	0.028247
		WETOs			
12h	257.32	6	42.89	1.286	0.305864
24h	7864.62	6	1310.77	2.829	0.035338
36h	25103.84	6	4183.97	2.423	0.061469
48h	39255.12	6	6542.52	2.761	0.038681

60h	104438.65	6	17406.44	3.228	0.020873
72h	141384.59	6	23564.10	2.989	0.028535
84h	231143.51	6	38523.92	3.117	0.024118
96h	244141.80	6	40690.30	2.349	0.068108
108h	351738.02	6	58623.00	2.632	0.046120

Table S12. Statistical analysis on the absolute deterrence index for males and females of *Sitona lineatus* L. (mean for all dates of observations) under EOTO and WETOs.

	Sum of Squares	df	Mean Square	F	p
		EOTO			
males	31103.10	3	10367.70	192.004	0.000000
females	29779.29	3	9926.43	61.901	0.000000
		WETOs			
males	12665.57	5	2533.11	4.642	0.001551
females	10471.35	5	2094.27	9.728	0.000002

Table S13. Statistical analysis on the number of aphids eaten by one 3-days larvae of *Harmonia axyridis* Pallas under EOTO.

Hours	Sum of Squares	df	Mean Square	F	p
6h	0.77778	2	0.38889	1.029	0.381124
18h	0.77778	2	0.38889	0.357	0.705464
30h	9.33333	2	4.66667	4.078	0.038530
42h	0.11111	2	0.05556	0.049	0.952314
54h	4.77778	2	2.38889	1.181	0.333862
66h	5.44444	2	2.72222	0.309	0.738777
78h	0.44444	2	0.22222	0.039	0.961788
90h	12.00000	2	6.00000	0.365	0.700121
102h	2.33333	2	1.16667	0.056	0.946015
114h	10.11111	2	5.05556	1.071	0.367604
Total	52.11111	2	26.05556	0.143	0.867939
Mean	0.52111	2	0.26056	0.143	0.867939
